# Association Between Intrapartum Papaverine Administration and the Risk of Obstetric Anal Sphincter Injuries: A Retrospective Cohort Study

**DOI:** 10.1007/s00192-026-06588-6

**Published:** 2026-04-15

**Authors:** Raneen Abu Shqara, Nadir Ganem, Ala Aiob, Maya Frank Wolf, Lior Lowenstein, Susana Mustafa Mikhail

**Affiliations:** 1 Raya Strauss Wing of Obstetrics and Gynecology Galilee Medical Center, Nahariya, Israel; 2 Azrieli Faculty of Medicine, Bar Ilan University, Safed, Israel

**Keywords:** Obstetric anal sphincter injury, Papaverine, Perineal trauma, Vaginal delivery

## Abstract

**Introduction and Hypothesis:**

Perineal trauma during vaginal delivery may result in long-term morbidity. We aimed to test the hypothesis that intrapartum administration of papaverine is associated with a lower incidence of obstetric anal sphincter injuries (OASIS) among primiparous patients.

**Methods:**

This retrospective cohort study included primiparous patients with singleton term (> 37 weeks) pregnancies who delivered vaginally at Galilee Medical Center, Nahariya, Israel, between March 2020 and June 2024. Patients who received intrapartum 80 mg intramuscular papaverine were compared with those who did not. The primary outcome was the incidence of OASIS, defined as third- (3A, 3B, 3C) and fourth-degree perineal tears. Secondary outcomes included the incidence of first- and second-degree perineal tears and episiotomy. Multivariable logistic regression identified independent predictors of OASIS.

**Results:**

Among 4939 patients, 1082 (21.9%) received papaverine. Baseline demographics of the two groups were comparable; however, patients who received papaverine had a higher BMI and a higher rate of labor induction. The incidence of OASIS was significantly lower in the papaverine group (0.5% vs 1.3%; adjusted absolute risk difference [aRD] −0.7%, 95% CI −1.2 to −0.1; *p* = 0.016). First- and second-degree perineal tears were less frequent in univariate analysis (35.2% vs 39.2%, *p* = 0.019); however, after multivariable adjustment, this association was attenuated and no longer statistically significant (aRD −1.3%, 95% CI −4.1 to 1.5; *p* = 0.37). Episiotomy rates of the two groups were comparable.

**Conclusions:**

In this study, primiparous patients who received intrapartum papaverine experienced a lower incidence of OASIS than those who did not receive papaverine. Further prospective, protocolized studies are needed to confirm these findings.

## Introduction

Perineal trauma refers to injury of the genital tissues that frequently occurs during vaginal childbirth. Among primiparous women, reported rates range from 35% to 78% for second-degree tears [1] and 0.7% to 2% for obstetric anal sphincter injuries (OASIS). In multiparous women, the corresponding rates are 35% to 40% and 0.1% to 0.3% respectively [2, 3]. Long-term complications of OASIS may include chronic pain, sexual dysfunction, urinary or fecal incontinence, and an increased risk of recurrent OASIS in subsequent pregnancies [4]. Reported risk factors for perineal trauma include previous perineal injury, prolonged second stage of labor, operative vaginal delivery, midline episiotomy, persistent occiput posterior presentation, nulliparity, fetal macrosomia, and maternal ethnicity [5–7].

Several intrapartum strategies aimed at improving perineal tissue compliance have been explored to reduce severe perineal trauma [8–10]. Papaverine hydrochloride is a smooth-muscle relaxant with vasodilatory properties that acts directly on smooth-muscle fibers and may enhance tissue elasticity during labor [11]. It has been used for decades in the treatment of vasospasm and spasms of the urinary and gastrointestinal tracts [12], and in obstetric settings has been associated with enhanced cervical ripening and shorter labor duration, without reported adverse maternal or neonatal effects [13–15]. The association between intrapartum papaverine administration and the incidence of OASIS has not been previously investigated. In contrast, several nonpharmacological approaches focusing on perineal management during the second stage of labor have been reported in the literature. In particular, active perineal protection techniques [16, 17] and the application of warm compresses during the second stage of labor have been associated with a reduced incidence of OASIS [17–21]. These interventions are thought to increase local tissue perfusion and elasticity, facilitating a more gradual stretching of the perineum, although causal mechanisms cannot be definitively established [17–21]. Despite its known smooth-muscle–relaxing and vasodilatory effects, it remains unclear whether papaverine confers any protective effect on perineal tissues or influences the risk of OASIS. We hypothesized that intrapartum papaverine administration is associated with a lower incidence of OASIS among primiparous patients. The primary aim of this study was to evaluate the association between papaverine administration during labor and the incidence of OASIS. A retrospective cohort design was used to explore this association in routine clinical practice, in the absence of protocolized use or available randomized trials.

## Materials and Methods

### Study Population and Setting

This retrospective cohort study included patients hospitalized at Galilee Medical Center, Nahariya, Israel, a tertiary care hospital affiliated with Bar-Ilan University, between March 2020 and June 2024. The study was approved by the Institutional Review Board (Helsinki Committee) of Galilee Medical Center (Approval No. 0010-25-NHR) and conducted in accordance with the Declaration of Helsinki.

The study population comprised term (> 37 weeks) primiparous patients with singleton pregnancies undergoing a trial of vaginal delivery. Exclusion criteria were multiparous patients, multiple gestations, pregnancies with significant congenital anomalies, and incomplete medical records. Patients with multiple papaverine doses were also excluded. Papaverine exposure was defined as documented administration of 80 mg intramuscular papaverine (IM) during labor, as recorded in the electronic medical records. In our department, papaverine may be administered at the discretion of the attending clinician during the latent phase of labor, typically when cervical dilation is less than 6 cm and the cervix is firm or noncompliant. Papaverine administration reflected routine clinical practice and was not implemented as part of a research intervention.

According to our labor ward protocol, all spontaneous low-risk vaginal deliveries are attended by midwives, whereas vacuum-assisted deliveries are performed by obstetricians. According to departmental protocol, during the expulsion phase, manual perineal support was provided using the “hands-on” technique [22, 23], which involved flexing the fetal head while supporting the perineum [24], and this practice remained unchanged throughout the study period. A restrictive policy toward mediolateral episiotomy was applied, with the incision performed at a 60° angle from the midline at crowning [25]. No midline episiotomies were performed.

### Outcomes

The primary outcome was the incidence of OASIS, classified according to the Sultan classification [26] (grades 3 A, 3B, 3 C, and fourth-degree tears), as adopted by international guidelines [8, 27]. Secondary outcomes included the incidence of additional perineal outcomes, such as first- and second-degree perineal tears and episiotomy. Other maternal and obstetric variables evaluated were maternal age, body mass index (BMI), gestational age at delivery, parity, diabetes mellitus, epidural analgesia, cervical dilatation, and Bishop score at admission, mode of delivery (vaginal or vacuum-assisted), induction of labor, duration of the second stage of labor, duration of rupture of membranes (ROM), birthweight (including macrosomia, defined as birthweight > 4000 g), and postpartum hemorrhage.

### Sample Size Calculation

In the absence of papaverine-specific data, the sample-size calculation was informed by a previous meta-analysis evaluating effective perineal protective interventions. An absolute risk reduction of 3.8% was considered clinically meaningful [28]. Assuming a two-sided α of 0.05 and 80% power, a total sample size of 762 participants would be required to detect a reduction in OASIS of a similar magnitude.

### Statistical Analysis

Continuous variables are presented as means ± standard deviations (SD) or medians with ranges, as appropriate. Categorical variables are presented as frequencies and percentages. Comparisons between patients who received papaverine and those who did not were performed using Student’s *t* test or Mann–Whitney *U* test for continuous variables, and the Chi-squared test or Fisher’s exact test for categorical variables. Baseline balance between groups was assessed using standardized mean differences (SMDs) for normally distributed continuous and binary variables. SMDs were interpreted using conventional thresholds: SMDs of 0.2, 0.5, and 0.8 are considered small, medium, and large respectively [29, 30]. Absolute risk differences (aRDs) were calculated for primary and secondary outcomes and reported as both unadjusted and adjusted estimates, with adjustment for maternal age, BMI, gestational week at delivery, induction of labor, vacuum-assisted delivery, Bishop score at admission, macrosomia, second-stage duration, rupture-of-membranes duration, and mediolateral episiotomy. Potential interaction between papaverine administration and vacuum-assisted delivery was examined using a logistic regression analysis that included an interaction term. A multivariable logistic regression analysis was performed to identify independent factors associated with OASIS, including variables found to be significant in univariable analysis or considered clinically relevant (maternal age, BMI, gestational week at delivery, induction of labor, oxytocin administration, balloon catheter insertion, vacuum-assisted delivery, Bishop score at admission, macrosomia, second-stage duration, ROM duration, and mediolateral episiotomy). Results are presented as odds ratios (ORs) with 95% confidence intervals (CIs). Model diagnostics included assessment of goodness-of-fit using the Hosmer–Lemeshow test. Multicollinearity among covariates was assessed using variance inflation factors (VIFs). Missing data for key variables were infrequent (< 1%) and were handled using complete-case analysis. All analyses were performed using IBM SPSS Statistics for Windows, version 27.0 (IBM Corp., Armonk, NY, USA). A two-sided *p* value < 0.05 was considered statistically significant.

## Results

### Baseline Maternal and Obstetric Characteristics

A total of 4939 patients were included, of whom 1082 (21.9%) received papaverine and 3857 (78.1%) did not. Among women receiving papaverine, most administrations occurred at 4-cm cervical dilation (573 out of 1082, 53.0%), followed by 5 cm (271 out of 1082, 25.0%) and 3 cm (238 out of 1082, 22.0%).

Patients who received papaverine were slightly older (27.5 ± 4.3 vs 26.6 ± 4.3 years, *p* < 0.001) and had a higher mean BMI (29.4 ± 4.8 vs 28.3 ± 4.5 kg/m^2^, *p* < 0.001). Obesity (BMI ≥ 30 kg/m^2^) was also more common in the papaverine group (436 [40.3%] vs 1234 [32.0%], *p* < 0.001). The mean number of pregnancies in the two groups was similar (*p* = 0.754), and the prevalence of diabetes mellitus did not differ significantly (*p* = 0.370). The rate of epidural analgesia use in the two groups was likewise comparable (*p* = 0.453). Gestational age at delivery was slightly higher in the papaverine group (39.6 ± 1.2 vs 39.5 ± 1.0 weeks, *p* < 0.001). At admission, patients in the papaverine group had a less favorable cervical status, with lower cervical dilatation (1.1 ± 1.1 vs 1.8 ± 1.5 cm) and Bishop scores (2.5 ± 1.9 vs 3.4 ± 2.2) compared with the comparison group (both *p* < 0.001). Induction of labor (783 [20.3%] vs 457 [42.3%], *p* < 0.001), oxytocin administration (1608 [41.7%] vs 705 [65.2%], *p* < 0.001), and balloon catheter insertion (303 [7.9%] vs 237 [21.9%], *p* < 0.001) were more common in the papaverine group. In contrast, the distribution of delivery modes (spontaneous vaginal vs vacuum-assisted) in the two groups was comparable (*p* = 0.939). The rate of macrosomia did not differ significantly between the groups. The duration of ruptured membranes was longer in the papaverine group (median 14.7 h vs 9.5 h, *p* < 0.001), whereas the duration of the second stage of labor was similar (*p* = 0.292). Among patients who received papaverine, the mean interval from administration to delivery was 8.8 ± 5.6 h (median 7.8, range 0.5–24 h). Based on SMDs, small baseline imbalances were observed for maternal age and BMI, and moderate imbalances were observed for induction of labor, oxytocin administration, balloon catheter insertion, Bishop score at admission, and cervical dilatation at admission. All other baseline characteristics demonstrated a good balance between the groups (Table 1).

**Table 1:** Maternal characteristics and obstetrical outcomes

Characteristic	No papaverine (*n* = 3857)	Papaverine (*n* = 1082)	SMD	*p* value
Maternal age (years), mean ± SD	26.6 ± 4.3	27.5 ± 4.3	0.20	< 0.001
BMI (kg/m^2^), mean ± SD	28.3 ± 4.5	29.4 ± 4.8	0.23	< 0.001
BMI < 25 kg/m^2^, *n* (%)	889 (23.0)	213 (19.7)	0.08	< 0.001
BMI 25–29.9 kg/m^2^, *n* (%)	1734 (45.0)	433 (40.0)	0.10	
BMI ≥ 30 kg/m^2^, *n* (%)	1234 (32.0)	436 (40.3)	0.19	
Pregnancy number, mean ± SD	1.2 ± 0.5	1.2 ± 0.6	0.02	0.754
Diabetes mellitus, *n* (%)	79 (2.0)	27 (2.5)	0.03	0.370
Cervical dilatation at admission, mean ± SD	1.8 ± 1.5	1.1 ± 1.1	−0.41	< 0.001
Bishop score at admission, mean ± SD	3.4 ± 2.2	2.5 ± 1.9	−0.45	< 0.001
Delivery week, mean ± SD	39.5 ± 1.0	39.6 ± 1.2	0.09	< 0.001
Epidural analgesia, *n* (%)	2822 (73.2)	804 (74.3)	0.03	0.453
Induction of labor, *n* (%)	783 (20.3)	457 (42.3)	0.50	< 0.001
Oxytocin administration, *n* (%)	1608 (41.7)	665 (61.4)	0.49	< 0.001
Prostaglandin E2 insert administration, *n* (%)	40 (1.0)	16 (1.5%)	0.02	0.255
Balloon catheter insertion for cervical ripening, *n* (%)	303 (7.9)	237 (2.2)	0.40	< 0.001
Delivery route, *n* (%)				0.939
Vaginal	3507 (90.9)	983 (90.8)	0.00	
Vacuum-assisted	350 (9.1)	99 (9.2)	0.00	
Birthweight (g), mean ± SD	3267.1 ± 405.2	3309.3 ± 453.3	0.10	0.003
Birthweight > 4000 g,, *n* (%)	128 (3.3)	48 (4.4)	0.06	0.080
Second stage duration (h), mean ± SD	1.1 ± 1.1	1.0 ± 1.1	0.05	0.292
ROM duration (h), median (range)	9.5 (0–110.9)	14.7 (0–143.3)	—	< 0.001
Postpartum hemorrhage,, *n* (%)	148 (3.8)	38 (3.5)	0.02	0.680

Missing data were < 1% for all variables

*BMI* body mass index, *ROM* rupture of membranes, *SD* standard deviation, *SMD* standardized mean differences

### Perineal Outcomes

For the primary outcome, the incidence of OASIS was lower among patients who received papaverine than among those who did not (5 [0.5%] vs 49 [1.3%], *p* = 0.024). After multivariable adjustment, papaverine administration remained associated with a lower adjusted absolute risk difference in OASIS (aRD −0.7%, 95% CI −1.2 to −0.1). Among patients who received papaverine, OASIS cases included 4 grade 3 A tears (0.4%) and 1 grade 3 C tear (0.1%); no grade 3B or fourth-degree tears were observed. In contrast, among patients who did not receive papaverine, OASIS included 23 grade 3 A tears (0.6%), 10 grade 3B tears (0.3%), 12 grade 3 C tears (0.3%), and 4 fourth-degree tears (0.1%). Any spontaneous perineal tear occurred less frequently in the papaverine group (386 [35.7%] vs 1559 [40.4%], *p* = 0.004), with a similar association after adjustment (aRD −4.4%, 95% CI −7.4 to −1.2). First- and second-degree tears were less common in unadjusted analyses (381 [35.2%] vs 1510 [39.2%], *p* = 0.019), but the adjusted absolute risk difference was not statistically significant (aRD −1.3%, 95% CI −4.1 to 1.5). Episiotomy rates in the two groups were similar both before and after adjustment (458 [42.3%] vs 1622 [42.1%], *p* = 0.871; adjusted aRD 0.7%, 95% CI −3.9 to 2.4) (Table 2).

**Table 2:** Perineal outcomes

Outcome	No papaverine (n = 3857)	Papaverine (*n* = 1082)	Unadjusted ARD, 95% CI	Unadjusted *p* value	Adjusted ARD, 95% CI*	Adjusted *p* value
Primary outcome						
OASIS, *n* (%)	49 (1.3)	5 (0.5)	−0.8 (−1.6 to −0.1)	0.024	−0.7 (−1.2 to − 0.1)	0.016
3A	23 (0.6)	4 (0.4)				
3B	10 (0.3)	0 (0)				
3C	12 (0.3)	1 (0.1)				
4	4 (0.1)	0 (0)				
Secondary outcomes						
All perineal tears, *n* (%)	1559 (40.4)	386 (35.7)	−4.7 (−7.9 to −1.5)	0.004	−4.4 (−7.4 to −1.2)	0.006
First- and second-degree perineal tear, *n* (%)	1510 (39.2)	381 (35.2)	−4.0 (−7.2 to −0.8)	0.019	−1.3 (−4.1 to 1.5)	0.037
Episiotomy, *n* (%)	1622 (42.1)	458 (42.3)	0.2 (−3.2 to 3.6)	0.871	0.7 (−3.9 to 2.4)	0.650

Missing data were < 1% for all variables

*ARD* absolute risk difference, *CI* confidence interval, *OASIS* obstetric anal sphincter injury

Adjusted for maternal age, BMI, gestational age at delivery, induction of labor, vacuum-assisted delivery, Bishop score at admission, macrosomia, second-stage duration, duration of rupture of membranes, and mediolateral episiotomy

### Interaction Analysis

No statistically significant interaction was observed between papaverine administration and vacuum-assisted delivery in relation to the risk of OASIS (interaction OR = 1.39, 95% CI 0.14–14.18; *p* = 0.780).

### Multivariable Analysis

In the multivariable logistic regression model (Table 3), papaverine administration remained independently associated with a lower risk of OASIS (OR 0.23, 95% CI 0.05–0.98; *p* = 0.046). Macrosomia and vacuum-assisted delivery were both independently associated with an increased risk of OASIS (OR 3.82, 95% CI 1.27–11.48; *p* = 0.017, and OR 5.38, 95% CI 1.81–15.98; *p* = 0.002 respectively). Mediolateral episiotomy demonstrated a significant protective effect (OR 0.24, 95% CI 0.10–0.60; *p* = 0.002). Other variables—including maternal age, epidural analgesia, induction of labor, oxytocin administration, balloon catheter insertion, Bishop score at admission, BMI, duration of ruptured membranes, and duration of the second stage—were not significantly associated with OASIS. The Hosmer–Lemeshow goodness-of-fit test indicated adequate model fit (Chi-squared = 7.498, df = 8, *p* = 0.484). All included covariates demonstrated low VIF values (approximately 1), indicating no problematic multicollinearity.

**Table 3:** Multivariable logistic regression analysis of factors associated with the incidence of obstetric anal sphincter injuries (OASIS)

Variable	Odds ratio	95% CI	*p* value
Papaverine administration	0.23	0.05–0.98	0.046
Epidural analgesia	1.62	0.69–3.78	0.264
Bishop score at admission	1.04	0.89–1.22	0.611
Maternal age	0.93	0.86–1.02	0.112
Oxytocin administration	0.60	0.28–1.27	0.182
Induction of labor	0.34	0.09–1.28	0.988
Balloon catheter insertion	1.16	0.29–4.58	0.835
Macrosomia	3.82	1.27–11.48	0.017
BMI	1.04	0.97–1.12	0.235
Mediolateral episiotomy	0.24	0.10–0.60	0.002
Duration of ruptured membranes (h)	0.96	0.83–1.09	0.431
Second stage duration (h)	0.81	0.58–1.12	0.954
Vacuum-assisted delivery	5.38	1.81–15.98	0.002

Missing data were < 1% for all variables

*BMI* body mass index, *CI* confidence interval, *OR* odds ratio, *ROM* rupture of membranes

## Discussion

### Main Findings

In this large retrospective cohort study of primiparous patients undergoing vaginal delivery, intrapartum administration of papaverine was independently associated with a significantly lower risk of OASIS. This protective effect persisted after adjusting for key clinical factors, including vacuum-assisted delivery and fetal macrosomia, both of which were identified as independent predictors of OASIS. Importantly, papaverine use did not increase the rates of episiotomy or other perineal trauma.

### Comment

Previous studies have demonstrated that papaverine enhances local microcirculation in both clinical and experimental models [31]. In patients with septic shock, intravenous papaverine transiently increased sublingual microvascular perfusion—specifically perfused vessel density and the microvascular flow index—without significant systemic hemodynamic effects [32]. Mechanistically, papaverine inhibits phosphodiesterases, increasing intracellular cAMP and cGMP, which reduces intracellular calcium, decreases myosin light-chain phosphorylation, and promotes actin depolymerization [9, 33–35]. Collectively, these mechanisms could plausibly enhance oxygen delivery and tissue elasticity, which may in turn reduce the likelihood of perineal trauma. In the present study, papaverine use was observationally associated with lower rates of OASIS and with a reduced incidence of total spontaneous perineal tears, potentially reflecting a broader protective effect on perineal integrity. Our findings are conceptually consistent with previous evidence supporting interventions that enhance perineal relaxation and elasticity—such as the use of warm compresses during the second stage of labor, which have been shown to reduce OASIS rates by up to two-thirds [17]. Although papaverine and warm compresses differ in mechanism (pharmacological vs thermal), their shared vasodilatory [35] and muscle-relaxant effects [9] may contribute to improved tissue compliance and gradual distension during crowning, thereby potentially minimizing sphincter overstretching [28, 36]. However, the present study was not designed to evaluate underlying mechanisms, and any potential mechanistic overlap remains speculative. Importantly, direct comparison with previous literature is limited, as no prior studies have specifically evaluated the association between papaverine or similar smooth-muscle relaxants and the risk of OASIS. Existing obstetric studies investigating the use of such agents have primarily focused on cervical ripening and labor progression rather than perineal outcomes [13, 15]. Notably, a randomized trial [15] evaluating papaverine before Foley catheter insertion demonstrated better Bishop score progression and shorter induction-to-delivery intervals than placebo. These findings highlight the capacity of papaverine to enhance smooth-muscle relaxation and cervical responsiveness, supporting the biological plausibility of similar effects on perineal tissue dynamics during childbirth.

Several labor-related factors may influence the risk of OASIS and warrant consideration when interpreting our findings. Uterotonic agents, including oxytocin and prostaglandin E_2_, are known to affect uterine and smooth-muscle contractility and may alter labor dynamics and perineal stretching [37, 38]. In our cohort, oxytocin use was more frequent among patients who received papaverine; however, it was not independently associated with OASIS after adjustment in the multivariable analysis. Prostaglandin E_2_ was used infrequently in both groups, limiting its contribution to multivariable modeling. Methods of labor induction, including balloon catheter insertion, may also influence perineal outcomes. In the present study, balloon catheter use was included in the multivariable model but was not independently associated with OASIS.

### Clinical and Research Implications

Further studies should explore whether the timing, dosage, or route of papaverine administration influences the observed association, and whether measurable changes in perineal perfusion or tissue elasticity can be demonstrated using objective imaging or elastographic techniques. Confirmation of these findings in well-designed prospective and randomized studies is required before any clinical recommendations can be made. Such studies would help to clarify whether papaverine has a reproducible role in perineal protection alongside established manual and thermal techniques used during the second stage of labor.

### Strengths and Limitations

This study is to our knowledge the first to suggest a potential protective effect of papaverine against OASIS. Although papaverine is traditionally used for cervical ripening, its impact on perineal outcomes has not been previously examined. A key strength of this analysis is its relatively large sample size, which enhances the robustness of the observed associations and provides a meaningful foundation for future investigation. These findings position papaverine as a novel, low-cost pharmacological adjunct for perineal protection and support the need for prospective, randomized studies to clarify its role. The study also has important limitations. The retrospective design and absence of randomization may introduce selection bias and confounding by indication, as papaverine administration was determined by clinician discretion rather than standardized criteria. Consequently, the decision to administer papaverine may have been influenced by underlying labor characteristics—such as perceived cervical rigidity, anticipated labor difficulty, or slower labor progress—that are not fully captured in the medical records. In addition, the two groups were not fully homogeneous at baseline; specifically, there were differences in Bishop score at admission, induction of labor, and maternal BMI between the groups. However, after adjustment in the multivariable analysis, none of these variables—including Bishop score, induction of labor, or maternal BMI—was independently associated with OASIS, reducing the likelihood that these baseline differences explain the observed association. Overall labor characteristics such as contraction strength, duration of labor, and progression patterns may contribute to perineal trauma. Provider-related factors, including the skill and experience of the birth attendant and delivery techniques, may also play an important role in minimizing perineal injury. These variables were not fully captured in the retrospective dataset and therefore could not be comprehensively evaluated. Moreover, the relatively small number of OASIS events in the papaverine group (*n* = 6) limits the precision of adjusted estimates. Given the low number of outcome events and the early timing of papaverine administration, conclusions regarding a specific biological mechanism should be considered exploratory, as the available data did not allow for a reliable evaluation of a distinct biological window of effect.

Although post-delivery perineal examination is routinely and systematically performed after all vaginal deliveries in our institution, underdiagnosis of OASIS cannot be completely excluded. The proposed biological mechanisms were not directly evaluated and remain speculative. Smooth-muscle relaxation may enhance tissue compliance but could also reduce natural perineal support, and accelerated cervical dilation may variably affect perineal strain. Accordingly, mechanistic interpretations and causal inferences should be made with caution. Finally, as this was a single-center study conducted within a specific labor ward setting, the findings may not be fully generalizable to other institutions with different clinical protocols, patient populations, or labor management practices.

## Conclusion

In this study, primiparous patients who received intrapartum papaverine experienced a lower incidence of OASIS than those who did not receive papaverine. Further prospective, protocolized studies are needed to confirm these findings.

## Data Availability

The data that support the findings of this study are not openly available due to reasons of sensitivity and are available from the corresponding author upon reasonable request.
